# Seasonal and regional presence of hymenopteran parasitoids of *Drosophila* in Switzerland and their ability to parasitize the invasive *Drosophila suzukii*

**DOI:** 10.1038/srep40697

**Published:** 2017-01-18

**Authors:** Valery Knoll, Thomas Ellenbroek, Jörg Romeis, Jana Collatz

**Affiliations:** 1 Agroscope, Biosafety group, Reckenholzstrasse 191, 8046 Zurich, Switzerland

## Abstract

Since its introduction into Europe the invasive *Drosophila suzukii* has established and spread widely, thereby entering habitats populated by native *Drosophila* species and their natural enemies. The highly prolific *D. suzukii* will likely interact with these species as a competitor, host or prey. To investigate potential interactions of *D. suzukii* with parasitoids, a field survey was conducted across several fruit-growing regions in Switzerland in two consecutive years. Eight species of hymenopteran parasitoids were collected using *D. melanogaster* as sentinel hosts in field-traps. Parasitoid capture was much higher in 2015 than in 2014 and varied among regions, time of the growing season, and habitat type. Laboratory no-choice assays with the field-collected species demonstrated that the larval parasitoids *Asobara tabida, Leptopilina boulardi,* and *L. heterotoma* could not use *D. suzukii* for reproduction, although the latter two reduced the number of emerging *D. suzukii*. In contrast, the pupal parasitoids *Pachycrepoideus vindemmiae*, *Trichopria drosophilae*, *Vrestovia fidenas* and *Spalangia erythromera* all developed with *D. suzukii* as hosts. Regional differences between strains were generally not evident, with the exception of two *T. drosophilae* strains that differed in parasitization rate. Thus, native parasitoids may interact with *D. suzukii* and should be regarded when implementing pest control measures.

Exotic insect species establishing in novel habitats interact with the living environment in their new habitat. The ecological consequences that arise from these interactions range from negligible effects to vast impacts on populations of other organisms^1^ and on ecosystem processes^2^. The newly established species may feed on plants or seeds, prey or parasitize upon other species and thus affect lower trophic levels^3^,^4^,^5^. However, they may also serve as prey or hosts to natural enemies^6^,^7^. In particular, rapidly multiplying, invasive herbivore species present an abundant resource to be exploited by predators and parasitoids that are able to include them into their prey or host range. Broadening host or prey range may occur immediately in preadapted natural enemy species or can take place after a phase of adaptation^6^. The consequences of these novel interactions depend on the acceptability and suitability of the herbivore as host or prey. Attractive but toxic preys or acceptable hosts that do not support adult parasitoid development may represent dead ends for the natural enemies and thus have negative consequences on their populations^8^,^9^,^10^. In contrast, a well-suited and abundant novel food source will allow the natural enemies to thrive and their enhanced populations may even reduce native herbivore populations via apparent competition^11^,^12^. Finally, management of invasive species may also result in ecological consequences when control efforts such as chemical or biological control also affect natural enemies^13^,^14^. Understanding the interactions of a novel herbivore species in an ecosystem is therefore a crucial prerequisite for the implementation of targeted control measures.

An invasive species that has received much attention recently is the spotted wing drosophila, *Drosophila suzukii* (Matsumura) (Diptera: Drosophilidae). It is an invasive frugivorous species native to Asia^15^, which causes considerable economic damage due to its ability to oviposit into intact ripening soft fruits^16^. Outside its native range it has been recorded for the first time in Spain, Italy, and in North America in 2008^17^,^18^ and within a few years it spread widely and established on both continents^19^. In Switzerland, the species has been detected for the first time in 2011^20^ and nowadays occurs across all fruit-growing areas^21^. It builds up large populations and adult individuals can be sampled throughout the year in agricultural and semi-natural habitats (ref. 22, own observations). *D. suzukii* entered ecosystems harbouring numerous native *Drosophila* species as well as their natural enemies. For example, in northern Switzerland twenty-five *Drosophila* species were identified at the edge of a single forest during a five month study^23^. Previous studies conducted in Italy^24^,^25^,^26^, Spain^27^, the United States^24^,^28^, and Mexico^29^ have found several native parasitoids to attack *D. suzukii* sentinel larvae and pupae deployed in the field. Thus, we assume that parasitoids of *Drosophila* present in Switzerland will accept *D. suzukii* as a host as well. Knowledge of the nature of the natural enemy interactions constitutes an important base for the development of control strategies. We thus studied potential interactions between *D. suzukii* and the local parasitoid community with the aims to (i) assess the parasitoid species composition in areas inhabited by *D. suzukii*, (ii) investigate the influence of habitat and time of the fruit growing season on parasitoid presence, and (iii) determine host quality of *D. suzukii* for the different parasitoid species and local strains in comparison to the common native host species *Drosophila melanogaster* Meigen and *Drosophila subobscura* Collin.

## Results

### Field samples

**Figure 1:**
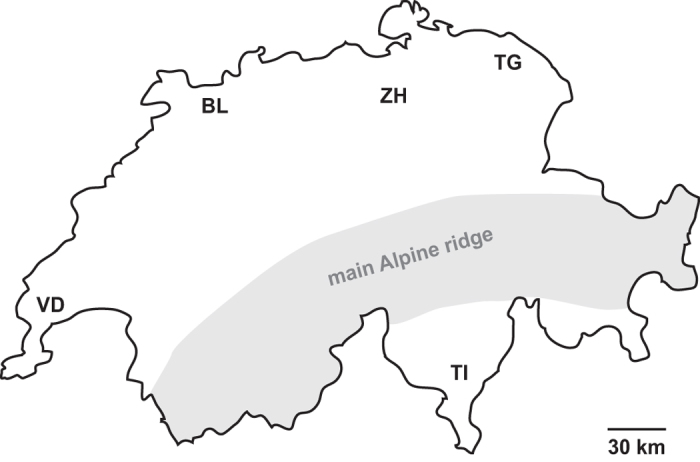
Outline of Switzerland indicating the four sampling regions. ZH: Zurich; TI: Ticino; TG: Thurgau; BL: Basel-Land; an additional strain of parasitoids was obtained from VD: Vaud. (The outline has been redrawn from http://d-maps.com/carte.php?num_car=2645&lang=de, using Adobe Illustrator CS6 16; 2012, www.adobe.com).

Eight species of parasitoids from four families of Hymenoptera emerged from the traps baited with *D. melanogaster* and set up in different locations in Switzerland (Fig. 1; Table 1). The most common parasitoid was *Pachycrepoideus vindemmiae* (Rondani), of which the highest overall number of individuals emerged and that was present in all regions. Also *Leptopilina heterotoma* (Thomson) was collected in all regions, while *Leptopilina boulardi* Barbotin *et al*. was not recovered from the northern-most region, Thurgau. *Trichopria drosophilae* (Perkins) was collected only south of the Alps in Ticino, whereas *Asobara tabida* (Nees) was only collected north of the Alps. *Trichopria modesta* (Ratzeburg) and the two pteromalid species *Vrestovia fidenas* (Walker) and *Spalangia erythromera* Förster were only collected occasionally. *D. suzukii* was present in both years during all three sampling time spans in all regions and in total 91% (2014) and 82% (2015) of fly traps contained adult *D. suzukii*.

**Figure 2:**
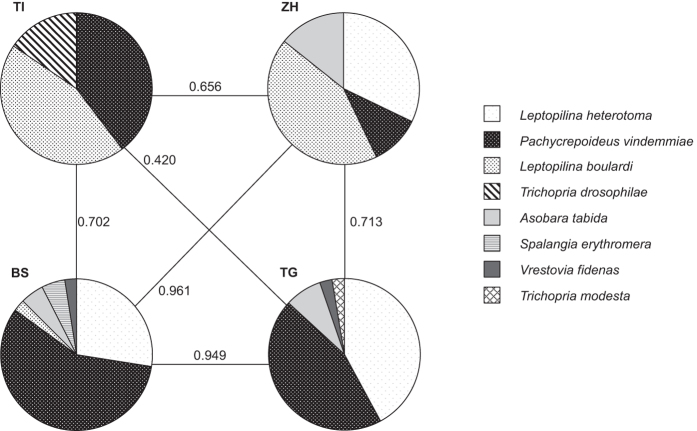
Parasitoid species incidence (measured as presence of parasitoid offspring) in *Drosophila melanogaster* infested fruit samples that had been exposed to parasitization by native *Drosophila-*parasitoids in the field in different regions in Switzerland (TI: Ticino; BL: Basel-Land; ZH: Zurich; TG: Thurgau) in 2015. N = 44–45 traps per region. Numbers and lines represent Chao-Sørensen-Raw Incidence based similarity indices comparing species composition between each two regions.

**Figure 3:**
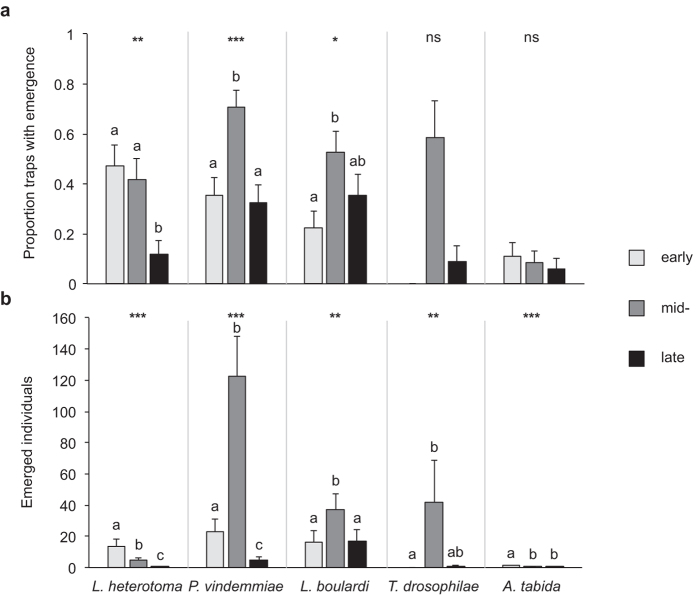
Influence of season on parasitoid presence in *Drosophila melanogaster* infested fruit samples that had been exposed to parasitization by native *Drosophila-*parasitoids in the field during early, mid- and late fruit growing season in Switzerland during 4 days in 2015. (**a**) Mean (+s.e.m.) parasitoid species incidence (measured as presence of parasitoid offspring), (**b**) Mean (+s.e.m.) number of parasitoids that emerged from samples. N = 11–12 traps per region per season, data pooled across regions: *L. heterotoma*: ZH, BL, TG; *P. vindemmiae*: ZH, TI, BL, TG; *L. boulardi*: ZH, TI, BL; *T. drosophilae*: TI, *A. tabida*: ZH, BL, TG. ***P < 0.001, **P < 0.01, *P < 0.05, GLM, different letters indicate significant difference, P < 0.05, Sequential Bonferroni post hoc test.

Species composition was similar in the three northern regions and differed most between Ticino and the northern regions (Fig. 2). Season had a significant influence on the incidence of *L. heterotoma* (generalized linear model GLM: *W*_2,102_ = 9.819, *P* = 0.007), *P. vindemmiae* (GLM: *W*_2,138_ = 16.492, *P* < 0.001) and *L. boulardi* (GLM: *W*_2,102_ = 7.025, *P* = 0.030) but not on *T. drosophilae* and *A. tabida* (Fig. 3). In contrast to the other species, *L. heterotoma* incidence was significantly higher during early and mid-season compared to late season, whereas *P. vindemmiae* was significantly higher during mid-season than during the two other seasons and *L. boulardi* incidence was higher during mid-season than during early season (Fig. 3a). A similar pattern was revealed when analysing the number of *L. heterotoma* individuals that emerged (GLM: *W*_2,102_ = 60.970, *P* < 0.001), *P. vindemmiae* (GLM: *W*_2,138_ = 182.770, *P* < 0.001), *L. boulardi* (GLM: *W*_2,102_ = 14.267, *P* < 0.001), *T. drosophilae* (GLM: *W*_2,32_ = 7.987, *P* = 0.005) and *A. tabida* (GLM: *W*_2,102_ = 19.882, *P* < 0.001). Most *L. heterotoma* emerged from early season samples, while emergence declined over the following two seasons. Likewise *A. tabida* emerged mainly from early season samples, with significantly fewer individuals collected later. *P. vindemmiae* emergence was highest in samples from mid-season and lowest in samples from late season. Also *L. boulardi* emergence was significantly higher in mid-season than during the two other seasons. *T. drosophilae* was only collected during mid- and late season with significantly more individuals emerging from the mid-season samples compared to the early season (Fig. 3b).

**Figure 4:**
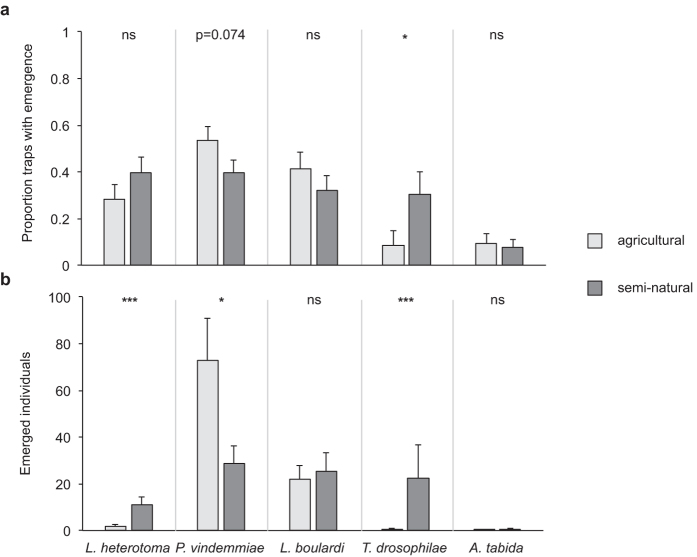
Influence of habitat on parasitoid presence in *Drosophila melanogaster* infested fruit samples that had been exposed to parasitization by native *Drosophila-*parasitoids in the field in agricultural and natural habitats in Switzerland during 4 days in 2015. (**a**) Mean (+s.e.m.) parasitoid species incidence (measured as presence of parasitoid offspring), (**b**) Mean (+s.e.m.) number of parasitoids that emerged from samples. N = 17–18 traps per region per habitat, data pooled across seasons and regions: *L. heterotoma*: ZH, BL, TG; *P. vindemmiae*: ZH, TI, BL, TG; *L. boulardi*: ZH, TI, BL; *T. drosophilae*: TI, *A. tabida*: ZH, BL, TG. ***P < 0.001, **P < 0.01, *P < 0.05, GLM, different letters indicate significant difference, P < 0.05, Sequential Bonferroni post hoc test.

Significantly more traps in semi-natural habitats contained *T. drosophilae* (GLM: *W*_1,32_ = 4.314, *P* = 0.038) than traps in agricultural habitats (Fig. 4a). *P. vindemmiae* (GLM: *W*_1,138_ = 3.186, *P* = 0.074) tended to occur more often in traps in agricultural habitats. Furthermore, significantly more individuals from *L. heterotoma* (GLM: *W*_1,102_ = 30.517, *P* < 0.001) and *T. drosophilae* (GLM: *W*_1,32_ = 21.635, *P* < 0.001) emerged from samples exposed in semi-natural habitats. More *P. vindemmiae* emerged from samples exposed in agricultural than in semi-natural habitats (GLM: *W*_1,138_ = 5.928, *P* = 0.015) (Fig. 4b).

### Parasitization assays

**Figure 5:**
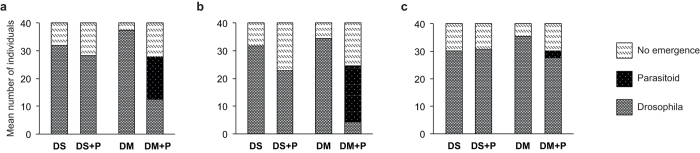
Mean number of fly and parasitoid individuals that emerged from each of 40 *Drosophila suzukii* (DS) and *Drosophila melanogaster* (DM) larvae that were exposed (+P) or not exposed to larval parasitoids for 5 days in no-choice parasitization assays. (**a**) *Leptopilina heterotoma* (strains: BL, ZH, and TG, n = 30), (**b**) *Leptopilina boulardi* (strains: BL and TI, n = 20), (**c**) *Asobara tabida* (strain: TG, n = 10).

The overall hatching rate of *D. suzukii* flies in the controls without larval parasitoids was 79.79 ± 1.21%. With the exception of two individuals of *L. heterotoma*, none of the three larval parasitoid species completed development on *D. suzukii* (Fig. 5). The number of emerged *D. suzukii*, however, was significantly reduced when larvae were exposed to *L. boulardi* (Basel-Land and Ticino-strains combined: GLM: *W*_1,36_ = 29.907, *P* < 0.001). In *L. heterotoma* the presence of parasitoids (GLM: *W*_3,72_ = 5.432, *P* = 0.020) as well as the factor strain (GLM: *W*_3,72_ = 12.194, *P* = 0.007) but not the interaction of both factors had a significant influence on the reduction of *D. suzukii* flies. The strain from Basel-Land reduced fly emergence significantly more than the strain from Ticino. *A. tabida* did not have any influence on the number of *D. suzukii* emerging.

**Figure 6:**
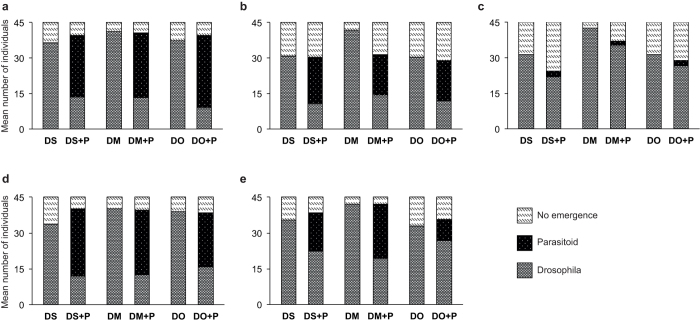
Mean number of fly and parasitoid individuals that emerged from each of 45 pupae of *Drosophila suzukii* (DS), *Drosophila melanogaster* (DM), and *Drosophila subobscura* (DO) that were exposed (+P) or not exposed to larval parasitoids for 5 days in no-choice parasitization assays. (**a**) *Pachycrepoideus vindemmiae* (strains: BL, ZH, TG, and TI n = 40), (**b**) *Spalangia erythromera* (strain: BL, n = 10), (**c**) *Vrestovia fidenas* (strain: TG, n = 10), (**d**) *Trichopria drosophilae* (strain: Vaud, n = 10), (**e**) *Trichopria drosophilae* (strain: TI, n = 10).

The overall hatching rate of *D. suzukii* flies in the controls without pupal parasitoids was 78.14 ± 1.40%. All tested pupal parasitoid species and strains reproduced on *D. suzukii* (Fig. 6). *P. vindemmiae* reduced *D. suzukii* emergence significantly (64.0% mean reduction; GLM: *W*_3,72_ = 21.370, *P* < 0.001) and produced a high number of offspring on pupae from all three host species offered. Parasitization indices did not differ significantly between host species or parasitoid strain (Table 2). *S. erythromera* reduced *D. suzukii* emergence significantly (62.3%; GLM: *W*_3,18_ = 80.648, *P* < 0.001) but produced overall fewer offspring than *P. vindemmiae* with parasitization indices being not significantly different between host species. Offspring production was lowest in *V. fidenas* and did not differ between host species, however also this species reduced *D. suzukii* emergence significantly (29.7%; GLM: *W*_3,18_ = 16.060, *P* < 0.001). In contrast, parasitization indices differed between parasitoid strains in *T. drosophilae* (Mann-Whitney-U-test: U: 192.5, *P* < 0.001), with higher offspring production in the strain from Vaud compared to the strain from Ticino. Parasitization indices did not differ between host species. Reduction of *D. suzukii* emergence was significantly influenced by parasitoid presence (GLM: *W*_3,36_ = 111.376, *P* < 0.001), parasitoid strain (strain Ticino: 37.0% and strain Vaud: 61.4% mean reduction; GLM: *W*_3,36_ = 19.297, *P* < 0.001) and the interaction of both factors (GLM: *W*_3,36_ = 13.385, *P* < 0.001) in *T. drosophila*.

## Discussion

Eight native parasitoid species were recorded in habitats recently invaded by *D. suzukii* in Switzerland. Parasitoid species composition differed among regions, in particular between Ticino in the south and Basel-Land, Zurich, and Thurgau north of the Alps, likely because of the different climatic conditions. Several species of *Drosophila* parasitoids seem to have their geographic distribution borders within Switzerland. For example, *T. drosophilae* had been recorded previously in Italy^26^,^28^ and Spain^27^ and was found during our surveys in Southern Switzerland in Ticino and Vaud (S. Fischer, Agroscope, pers. comm.), however, it was not retrieved from any of the samples from northern Switzerland. Like Central France, Switzerland seems to be at the northern geographic limit of *L. boulardi*, a species native to the Mediterranean region that is currently expanding its distribution range northwards^30^. Previous studies have shown that developmental success of *L. heterotoma* is reduced in the presence of *L. boulardi*, but not *vice versa* and in cage experiments *L. boulardi* outcompeted *L. heterotoma*^31^. However, differences in overwintering strategies with *L. heterotoma* being active several weeks before *L. boulardi* mediate the coexistence of the two species^32^,^33^. *L. boulardi* was found in Ticino in both study years, in Zurich and Basel-Land only in 2015, but was never collected in Thurgau. It is possible that in a year with high average temperatures *L. boulardi* is able to build up large populations in northern Switzerland and limit reproduction for *L. heterotoma* from mid-season on. This idea is corroborated by the observation that incidence and abundance of *L. heterotoma* decreased from spring to fall. It might be also valid for the larval parasitoid *A. tabida*, which also interacts with the two *Leptopilina* species^32^ and of which the number of emerged parasitoids decreased over the course of the growing season.

In contrast to *L. heterotoma* and *A. tabida*, the number of emerged parasitoids was highest in summer for *L. boulardi*, *T. drosophilae* and *P. vindemmiae*. This observation is in accordance with findings from northern Italy^26^,^28^ and might be explained by a combination of fluctuations of the parasitoid populations in the course of the season and a thermally induced high activity of parasitoids that leads to higher trap captures. In all regions the number of parasitoid species and individuals collected in 2015 was markedly higher than in 2014. It is likely that the differences can be attributed to the different weather conditions. In 2014 a particular mild winter was followed by a cool and humid summer, both favourable conditions to the build-up of large *D. suzukii* populations^34^,^35^. This is evident from the important economic damage incurred by Swiss fruit growers in 2014^36^. Additionally the high humidity led to ruptures and fungal infections of the fruits, thus fostering native *Drosophila*-species as well. As natural enemy populations respond to an increase in host populations with a time lag, our sentinel traps competed with a high natural host availability. In contrast, a high number of parasitoids was present after hibernation in 2015, when hot and dry summer conditions reduced host availability and this might have resulted in the remarkably high emergence of parasitoid offspring from traps in 2015.

In particular *P. vindemmiae* was present in a very high proportion of traps. It was also the only parasitoid, for which significantly higher emergence was recorded from traps set up in agricultural habitats compared to traps in the semi-natural habitats. It seems that this parasitoid copes well with high temperatures and dry conditions as it prevailed in most of the agricultural habitats during summer. This species may play a particular role in shaping the communities of flies and parasitoids in Switzerland as it interacts with them in several ways. First, *P. vindemmiae* is a broad generalist on numerous *Drosophila* species including *D. suzukii*^37^,^38^ as well as other Dipterans^39^. It is thus likely to benefit from the new and abundant resource that is provided by the invading *D. suzukii*. Enhanced populations of the parasitoid may then affect other fly species via apparent competition. Second, *P. vindemmiae* can hyperparasitize *Leptopilina* spp. and *Asobara* spp^40^. Therefore, its high abundance in agricultural habitats may limit the numbers of the larval parasitoids in the same habitat via intraguild predation. Third, it may also compete for host resources with other pupal parasitoids^41^.

Often, semi-natural habitats provide more suitable microclimates and alternative food and host resources to parasitoids compared to agricultural habitats^42^. This may be the case for *L. heterotoma*, *A. tabida,* and in particular for *T. drosophilae*, which was found almost exclusively in the semi-natural habitats. The latter species seems to be active only from mid-season on (ref. 26 and Fig. 3), despite having an upper thermal limit for adult survival at around 34 °C^43^, a temperature that is often reached in Switzerland in open field conditions during summer. In particular for this species thermal limitations in the crop environment as well as an activity peak late in the growing season may reduce the impact on *D. suzukii* populations that are active in Switzerland even during the winter^22^. Thus, targeting the microhabitat structure as well as enhancing parasitoid populations early in the growing season could be promising strategies in the biological control of *D. suzukii*. In a recent publication, the relevance of unmanaged, semi-natural habitats has been also pointed out by Wang *et al*.^44^. These habitats may play an important role for the population dynamics of *D. suzukii* as they can serve as reservoirs for the recolonization of crops after the application of insecticides and provide shelter for the flies during unfavourable weather conditions^45^,^46^. Further studies are required to investigate their relative importance for *D. suzukii* and the community of potential natural enemies to assess the impact of management actions.

Our laboratory assays demonstrate for the first time the ability of the pupal parasitoids *S. erythromera* and *V. fidenas* to utilize *D. suzukii* as hosts. Together with the already reported *P. vindemmiae* and *T. drosophilae*^27^,^38^, they add to the community of species that could play a role in the control of *D. suzukii.* While parasitization rates varied largely among species, with most parasitoids emerging in *P. vindemmiae* and fewest in *V. fidenas*, all pupal parasitoids developed on *D. suzukii* at a rate comparable to that on the other two native *Drosophila* species tested. No major strain differences in parasitization of *D. suzukii* were detected between *P. vindemmiae* from different locations in Switzerland. Likewise, Chabert *et al*.^38^ could not detect any difference between strains collected from different locations within France, whereas differences were detected between strains from the United States and strains from Italy^25^. In our study *T. drosophilae* from Vaud produced significantly more offspring than a strain from Ticino, likewise strains from the United States and South Korea and from different areas within France differed in their parasitization efficacy on *D. suzukii*^38^,^44^. Thus, a careful evaluation of the biological characteristics of the used strain is crucial to the evaluation of this species as a potential biocontrol candidate for *D. suzukii*.

In contrast to the pupal parasitoids, the larval parasitoids *L. heterotoma*, *L. boulardi* and *A. tabida* were not able to develop on *D. suzukii* in our study, although numbers of emerged flies were significantly reduced for *D. suzukii* in the presence of *L. boulardi* and *L. heterotoma*, probably due to unsuccessful parasitization events. Even stronger than *D. melanogaster*, *D. suzukii* defends itself against larval parasitoids by encapsulating and subsequent melanising the parasitoid eggs within the larval tissue^47^,^48^. However, the immune response of the flies seems to be costly, as it is associated with a reduced feeding rate^49^ and a reduced fecundity of the surviving adults^50^,^51^,^52^. Furthermore there appear to be indirect fitness costs as *P. vindemmiae* has been found to preferentially parasitize pupae of hosts that had been attacked as larvae by *A. tabida*^53^. Consequently, the presence of these species may still have a negative impact on *D. suzukii* populations.

Novel invasive species such as *D. suzukii* can act as a sink for the native parasitoid populations, when eggs are deposited into the unsuitable hosts. Such an ecological trap^54^ provided by an invasive species has been observed for the egg parasitoid *Telenomus podisi* Ashmead (Hymenoptera: Scelionidae) that accepts the non-native *Halyomorpha halys* (Stål) (Hemiptera: Pentatomidae) for oviposition but is not able to develop within this host^10^. Abram *et al*.^10^ suggest that reduced abundance of *T. podisi* due to the presence of *H. halys* could also cause a release from control for native pentatomid species in the sense of apparent predation (*sensu* Holt^11^).

Outcomes of the interactions between larval parasitoids and *Drosophila* spp. also depend on the specific strains of parasitoids and flies involved^55^,^56^,^57^. Within the Swiss populations of *L. heterotoma* differences in lethality to flies were observed among strains. Strain-specific differences could also explain discrepancies between our results and other studies. For example a study from Italy found a high mortality in *D. suzukii* larvae exposed to *L. heterotoma* but not in those exposed to *L. boulardi*^26^. Another study even observed development and emergence of *L. heterotoma* on *D. suzukii*^25^.

However, in how far the parasitoids attack *D. suzukii* under natural conditions remains to be investigated for most of the species. In particular for the larval parasitoids, studies with a long exposition time of samples in the field bear the risk of misinterpretation when native *Drosophila* were able to lay additional eggs in the bait and larvae hatching from those eggs then became parasitized. This aspect is less likely for the observation of the pupal parasitoids *P. vindemmiae* and *T. drosophilae* that were recovered from *D. suzukii* in the field^27^,^25^. While many pupal parasitoids are known to develop on *D. suzukii* under controlled laboratory conditions and in no-choice assays, little information is available on the host-finding and host-choice behaviour in the presence of native *Drosophila* species. To our knowledge, only two studies have addressed this aspect by the investigation of *T. drosophilae*, which has been shown to produce a higher number of offspring on *D. suzukii* in a choice situation with *D. melanogaster*^26^,^44^. Differences in offspring numbers were attributed to a higher mortality of the parasitoid in *D. melanogaster* pupae rather than to a host-preference^26^.

We conclude that a complex of native parasitoids potentially interacts with the invasive *D. suzukii* in agricultural and semi-natural habitats in Switzerland, and likely in other temperate regions of Europe. The diversity of the parasitoids, their biological characteristics and their requirements provide the potential for enhanced pest control, in particular via improvement of the habitat. As the interactions among native and exotic flies with their natural enemies can be manifold, care has to be taken when implementing control measures against *D. suzukii*, as these might affect multiple species on several trophic levels.

## Material and Methods

### Insects

Cultures of *D. suzukii* and *D. subobscura* originated from individuals collected in Zurich-Affoltern, Switzerland in 2013. *D. melanogaster* were obtained from a laboratory wild-type culture from Professor Walter J. Gehring’s Lab (University of Basel, Switzerland). Larvae of all *Drosophila* species were reared on an artificial diet based on banana (400 g Banana, 20 g agar-agar, 50 g brewer’s yeast, 30 g wheat flour, 20 g saccharose, 4 g nipagin, 1 l water) within plastic jars (11 cm dia., 15 cm height) sealed with a plastic gauze that allowed gas exchange. Upon emergence, adults were transferred either into flight cages (*D. melanogaster*, *D. subobscura*, 32 * 22 * 16 cm) or into plastic jars (11 cm dia., 15 cm height) with a fine metal grid at the bottom, which were placed onto the artificial diet within a plastic cup (*D. suzukii*). Adults in the flight cages were allowed to oviposit directly onto fresh blocks of diet, while adults in the jars oviposited through the metal grid. The diet was exchanged three times per week and adult flies were replaced every four weeks. All rearings were kept in climate chambers at 70% RH, 14:10 L:D and 22 °C (*D. suzukii, D. subobscura*) or 25 °C (*D. melanogaster*).

Parasitoids of *Drosophila* originated from field collections as described below; an additional strain of *T. drosophilae* was collected in Vaud. Cultures were kept separated by strain in flight cages (32 * 22 * 16 cm) at 22 °C, 70% RH, 14:10 L:D. Larval parasitoids were reared on larval stage 1–2 of *D. melanogaster (L. boulardi* and *L. heterotoma*) or *D. subobscura (A. tabida*). Therefore, diet from the fly rearing that was infested with *Drosophila* larvae was supplemented with fresh diet and exposed to the parasitoids for 3–4 days. Pupal parasitoids were reared on pupae of *D. melanogaster (T. drosophilae, P. vindemmiae, V. fidenas, S. erythromera*). Pieces of cotton wool (Dental rolls, 12 mm dia., Gerber Instruments, Effretikon, Switzerland) were introduced into the *Drosophila* rearing jars when larvae were close to pupation and removed after 24 h. The cotton wool with freshly formed pupae was then exposed to the parasitoids for 3–4 days. Parasitized larvae and pupae were transferred into cups sealed with a plastic gauze (6 cm dia., 8 cm height) and kept until emergence of parasitoids 3 to 5 weeks later. Four to 7 days after emergence, parasitoids were either used for parasitization experiments or introduced into the flight cage for further rearing.

### Field sampling

Four major fruit-growing regions in Switzerland (Cantons: Ticino, Zurich, Thurgau, and Basel-Land) were chosen for field sampling (Fig. 1). In each region, 12 locations were sampled, of which 6 were agricultural sites with fruiting crops and 6 were semi-natural sites, either forest or hedgerows. Fruiting crops comprised according to season cherry, berries, plum and grapevine, all of which are suitable and common hosts for *D. suzukii*. In each region sampling was conducted once during early, mid- and end of growing season (2014: 23.6.−26.7., 11.8.−12.9., 15.9.−11.10; 2015: 08.6.−10.7., 27.7.−21.8., 7.9.−2.10.) resulting in a total of 144 samples per year. While locations were kept the same for semi-natural sites during the growing season, agricultural locations were chosen to provide available host fruits in a stage susceptible to *D. suzukii* attack and thus had to be changed between the sampling time spans. Traps were deployed for 4 days but baits were exchanged after 2 days to avoid desiccation and to provide appropriate host stages to the parasitoids.

To assess the presence of *D. suzukii* a custom-made cylindrical fly-trap (9.5 cm dia., 12 cm height) with 10 entry holes (4 mm) baited with 250 ml of a mixture of red-wine, vinegar, water (1:1:1), and a few droplets of detergent was placed at a distance of approx. 5 m from each parasitoid-trap.

#### Traps

To allow for parasitoid oviposition plastic dishes (6 cm dia.) with diet (only 2014) or seasonal fruits (cherries or plums) that had been pierced multiple times with a needle to enable fly access were exposed for 48 h to adult *D. melanogaster* inside a flight cage at 25 °C. Baits were prepared either directly (larval stage 1–2) or after another 48 h (beginning of pupation) using either 25 g infested diet, 3 cherries, or ½ plum. Self-constructed plastic Delta-traps (20*20*10 cm; white, with red edges) were baited with larvae and pupae (modified after Rossi-Stacconi *et al*. 2013) as well as with approx. 15 caged 2–4-day old adult *D. melanogaster* of both sexes on artificial diet, hence taking into account the role of adult fly pheromones in host location of the parasitoids^58^. In 2014, each Delta-trap contained two dishes with diet and two dishes with seasonal fruits; in 2015 traps were modified to a smaller size (20*13*8 cm) and contained only two baits with seasonal fruits. For each collection date, one bait sample was kept in the laboratory to assess the number of emerged flies.

#### Parasitoid collections

Samples from the field were kept in plastic jars (9.5 cm dia., 12 cm height) that were sealed with a plastic gauze to allow gas exchange within a walk-in climate chamber (22 °C, 70% RH, 14:10 L:D) for six weeks. Samples were checked three times a week and emerged flies and parasitoids were collected using an aspirator. Number and species of emerged parasitoids per sample were recorded. The number of emerged parasitoid offspring from a sentinel bait is proportional to the number of female parasitoids attracted to the sample. Thus, counting emerged parasitoids overestimates true effects, while determining solely presence/absence of a species underestimates true effects. We report both values, as the number of eggs laid by each female is unknown and affected by various factors such as the species, the physiological status and the time spent at the trap.

Collected parasitoids were used to establish laboratory cultures, while small subsamples were preserved in Eppendorf-vials with 70% ethanol as voucher specimens. Figitidae parasitoids were identified using the taxonomic keys published by Nordlander^59^ and Forshage and Nordlander^60^. Pteromalidae, Diapriidae and Braconidae were identified by taxonomic experts.

#### Parasitization assays

Parasitoid species and strains were comparatively tested for their ability to parasitize *D. suzukii, D. melanogaster* and *D. subobscura* (only pupal parasitoids) in no-choice assays. Prior to the assays newly emerged male and female parasitoids were kept with a droplet of honey but without hosts for 4–7 days to assure mating. For larval parasitoids, 40 first instars of either *D. suzukii* or *D. melanogaster* were placed on a block of diet (2 g) with a fine metal hook under a stereo microscope. The diet was then transferred into a vial (2 cm dia., 6 cm height) containing a humid piece of cotton wool and a droplet of honey. The vial was sealed with a foam plug that allowed gas exchange. For pupal parasitoids, a paper tissue was added to the rearing jars of *D. suzukii, D. melanogaster* and *D. subobscura* 24 h prior to assays to provide the larvae a substrate for pupation. Subsequently, the paper was cut into pieces containing 45 freshly formed pupae that were then introduced into a ventilated vial (6 cm dia., 8 cm height). A piece of humid cotton wool and a droplet of honey were added. Single female parasitoids were added to half of the vials, whereas the other half served as control. Ten replicates were performed per parasitoid strain and host species. No more than five replicates were conducted during the same week to assure that all parasitoids and hosts originated from at least two different batches.

### Statistical analysis

From the total of 288 traps, three were excluded from the final analysis because they had been vandalized. Due to the low number of collected parasitoids in 2014, we focused detailed statistical analysis for field collections on data from 2015.

Chao’s Sørensen similarity indices for replicated incidence based data^61^ were calculated for comparison of parasitoid species composition among regions using estimateS (Version 9, 2013, Robert K. Colwell). All other statistical analyses were performed using SPSS, version 23 (IBM Corp., Armonk, New York, 2015).

To investigate the influence of the factors season and habitat on the five most prevalent parasitoid species (*L. heterotoma*, *L. boulardi*, *P. vindemmiae*, *T. drosophilae*, and *A. tabida*), data were pooled across regions. Only regions were included into the analysis, where the respective parasitoid species had been collected. Generalized linear models (main-effect) were applied to investigate the effects of habitat and season on a) the incidence of parasitoid species, i.e. the number of traps that contained at least one individual of a particular species, and b) the abundance of parasitoids, i.e. the number of parasitoid individuals of a particular species that emerged from a trap. As a preliminary analysis did not detect significant differences for any of the species between the habitats “hedges” and “forests” these habitats were pooled as semi-natural habitats. Therefore the final models contained the parameters “early”, “mid-“ and “late” for the fixed factor “season” and the parameters “agricultural” and “semi-natural” for the fixed factor “habitat”.

The binomial data on incidence were modelled with binomial logistic error distribution and count data on abundance were modelled with negative binomial error distributions due to over-dispersion of errors when assuming Poisson distribution. Where significant differences for the factor season were detected, post-hoc tests were conducted with sequential Bonferroni corrections.

In the laboratory parasitization assays, the effect of parasitoid presence and, where applicable, of parasitoid strain and the interaction of both factors on the number of emerged *D. suzukii* was analysed using generalized linear modelling. Poisson or negative binomial error distribution were used according to model fit (Omnibus-test; ratio: deviance/df). Where significant strain differences were detected, post hoc tests with sequential Bonferroni corrections were used for pairwise comparisons of strain effects.

As controls demonstrated differences in survival rates of unparasitized pupae of the three fly species, a parasitization index was calculated, i.e., the ratio between emerged parasitoids and total emerged individuals (flies + parasitoids) to compare the level of successful parasitoid development of pupal parasitoids on different host species. Due to heteroscedasticity indices were compared between host fly species and parasitoid strain using non-parametric Kruskal-Wallis-tests. The data are stored at DOI 10.6084/m9.figshare.4054731.

## Additional Information

**How to cite this article**: Knoll, V. *et al*. Seasonal and regional presence of hymenopteran parasitoids of *Drosophila* in Switzerland and their ability to parasitize the invasive *Drosophila suzukii. Sci. Rep.* **7**, 40697; doi: 10.1038/srep40697 (2017).

**Publisher's note:** Springer Nature remains neutral with regard to jurisdictional claims in published maps and institutional affiliations.

## Figures and Tables

**Table 1:** Regions, number of emerged parasitoid individuals and number of traps with parasitoid emergence from field samples in 2014 and 2015.

Family, Species	Regions	Individuals	Traps
**Braconidae**			
*Asobara tabida*	ZH, TG, BL	58	9
**Diapriidae**			
*Trichopria drosophilae*	TI	520	9
*Trichopria modesta*	TG	4	1
**Figitidae**			
*Leptopilina boulardi*	ZH, TI, BL	2498*	39*
*Leptopilina heterotoma*	ZH, TI, BL, TG	695*	36*
**Pteromalidae**			
*Pachycrepoideus vindemmiae*	ZH, TI, BL, TG	7585	82
*Spalangia erythromera*	BL	62	2
*Vrestovia fidenas*	BL, TG	13	2

ZH: Zurich; TI: Ticino; TG: Thurgau; BL: Basel-Land.

^*^2014: A total of 1836 *Leptopilina* sp. emerged from 17 traps.

**Table 2:** Parasitization indices (Mean ± s.e.m.) of pupal parasitoids in no-choice assays when offered 45 host pupae during 5 days.

	strains	*D. suzukii*	*D. melanogaster*	*D. subobscura*
*P. vindemmiae*	**BL, ZH, TG, TI**	0.65 ± 0.06	0.66 ± 0.06	0.74 ± 0.06
*S. erythromera*	**BL**	0.62 ± 0.04	0.53 ± 0.08	0.58 ± 0.07
*V. fidenas*	**TG**	0.13 ± 0.07	0.06 ± 0.03	0.11 ± 0.06
*T. drosophilae*	**Vaud**	0.68 ± 0.07	0.68 ± 0.08	0.60 ± 0.05
*T. drosophilae*	**TI**	0.45 ± 0.06	0.53 ± 0.07	0.30 ± 0.06

Parasitization indices represent the number of emerged parasitoids divided by the number of flies + parasitoids emerged.
